# Some Diarrheal Diseases of Children1Read before the Medical Society of the County of Erie, January 11, 1898.

**Published:** 1898-03

**Authors:** F. H. Stanbro

**Affiliations:** Springville, N. Y.


### SOME DIARRHEAL DISEASES OF CHILDREN.[1]

By F. H. STANBRO, M. D., Springville, N. Y.

IT IS not my purpose today to read an exhaustive paper on diarrheal diseases of infancy and childhood, but only to touch lightly on certain points of treatment of a few of the more common troubles that we see in this section of the country. So much has been written, so many experiments have been made and so much pathological work has been accomplished, that theories and divisions have no interest for us today, except as they appeal to us as clinicians. 'Tis said by a writer on children's diseases, Holt, I think, that 95 per cent. of diarrheal diseases in children occurs in bottle-fed children. This may seem an exaggerated statement, but it is true in the main in the country and may be wholly true in the city.

Mother's milk is the ideal baby food and I formerly insisted on every mother nursing her baby, but after two lamentable failures, one of which resulted in death of the child, I now examine the milk of a mother whose baby is not thriving and if it shows a condition that cannot be remedied, I wean the infant.

I have had no success with the artificial so-called "foods" of the market, but have used successfully cow's milk, when properly diluted and modified. It is best diluted with barley water, oatmeal water or lime water, to the proper density, and sweetened with cane sugar, not neglecting to add salt. Infants need salt and sugar in food just as they need water to allay thirst. Because their diet is liquid we must not judge they do not need water. We all have seen irritable, feverish, crying babies drink ravenously of cold water and drop off into a quiet, peaceful sleep. Jacobi says there is no virtue in lime water, but it has always worked well

1. Read before the Medical Society of the County of Erie, January 11, 1898.

in my practice ; it may be on account of the dilution and not from the virtue of the alkali.

From my observation, a thing much neglected in treating diarrheas of children is the daily examination of the diapers of the sick babies. The mother's description may be very vague ; she may give a wrong impression entirely ; fat may seem to her to be curds and vice versa and the color sense is poorly cultivated among the laity ; she cannot be expected to distinguish between bile and blood changed by the intestinal juices. And especially in these cases of artificially-fed babies, I deem it of great importance to watch the stools, the appearance of which will give so many hints for changes in care and treatment. Curds in the stools demand greater dilution, lumps of fat decrease of cream, and so forth.

There are two or three kinds of diarrheal diseases that we see constantly during the summer months, which differ in cause, course and symptoms, as well as treatment and prognosis. The one that causes as much sickness and as many puny rachitic babies, if not as many deaths, as any other, is the acute dyspeptic diarrhea, as described by Osler and others. As the name implies, this class of trouble is caused by either fermented or improper food or a lowering of the digestive power of the infant through previous illness, atmospheric heat or general weakness, causing imperfect or delayed digestion. The undigested food acts as an irritant and nature's efforts toward health causes the diarrhea. The child seems in usual health, except restlessness, slight attacks of colic and crying, an increase in number of stools, which are normal in appearance, except presence of undigested curds— if the diet is milk—or other undigested food. Also, the stools are extremely acid and excoriate the surrounding parts and have a sour odor.

If properly treated in this stage, by a dose of castor oil, syrup of rhubarb, calomel or the milder gray powder, the intestines will be cleansed of the irritating material and with a change to proper diet all is well. In fact, it is a mild affair and often passes by without the calling of a physician, unless the improper feeding is continued, when the symptoms grow worse, vomiting occurs, the temperature rises, the stools become more profuse and contain more fluid and tenesmus appears with mucus and blood, showing a general involvement of the intestinal tract.

At the beginning of vomiting the physician is always called, as that is the nervous mother's sign of cholera infantum. There are three indications for treatment: first, empty the intestines, disinfect the intestines, then correct the diet. If vomiting is constant get the stomach quieted before giving any laxative, although 1-10 grain tablet triturates of calomel sometimes seem to act as a sedative and laxative too. The multitude of drugs advised to restrain vomiting proves the unreliability of them all and in my practice I have given them all up and only order a withholding of all nourishment, even water except in very small quantities, for six, twelve and even twenty-four hours. When the vomiting ceases, I begin to give albumin water by the spoon, gradually working back to a normal diet. For the disinfection of the intestines, calomel, 1-10 grain doses, four to six times a day and continued for a few days, works favorably. Salol has proven of much value and Jacobi recommends naphthalin and resorcin very highly. Astringents, such as tannic or gallic acids, combined with large doses of bismuth subnitrate have been of value in my cases, but the remedy which has been of the greatest aid to me is arsenite of copper. There is no drug which appears to help so many physicians and again fails to have any effect in the hands of others as this drug, but, in my hands, dissolving 1-100 grain tablet in water, four ounces, giving 1 drachm doses every fifteeen minutes, until an hour or two has passed, then continuing the dose every hour, gives good results. The stools improve in character, their number is lessened and griping ceases within twelve hours.

In the same children, which have several attacks of dyspeptic diarrhea during a season, appears the more dangerous and startling cholera infantum. This disease sets in in the hottest weather. Seibert claims that atmospheric heat bears a direct influence on diarrheal diseases, and humidity and barometric pressure no influence. Diarrheal troubles begin to increase in the latter part of May, reach the maximum in August and September and decrease as cooler weather comes on. We have all noticed how a hot spell increases the number of cases of this kind and, again, how a sudden cool wave will benefit the little sufferers.

Without doubt cholera infantum is of germ origin, although no constant organism has yet been found. No warning is given in this disease; the onset is sudden, the collapse extreme; vomiting constant and exhaustive; stools profuse, watery, serous and numberless, at first very offensive, but later become odorless and fade into the napkin, leaving only a slight stain, as though the child had urinated; temperature rises very high, pulse feeble and rapid; from being restless at first the child passes into stupor.

This is the class of cases that needs sustaining and stimulating treatment, which the constant vomiting hinders, if it does not prevent. Washing out the stomach sometimes relieves the vomiting and if large quantities of water are given in vomiting the stomach will wash itself. High injections of cold water will relieve high temperature, but children do not bear cold well and hot water will be absorbed and act as a stimulant, especially if rendered alkaline by the addition of some salt. Astringents or opium may be given by rectum, but it is better to give morphine hypodermatically; a child one year old may be given 1-80 to 1-40 grain every hour or two for a few doses with safety and benefit. Brandy or champagne given cold may help settle the stomach and control collapse, and if diluted may be used hypodermatically or per rectum.

The advice of the best authors is to sustain the patient by combatting collapse with external heat and hot baths, giving strychnine nitrate hypodermically, with the addition of morphine for its effect on the bowels and sustaining effect on the heart. If the stomach will retain anything, champagne and such diffusible stimulants as camphor, ammonia or musk may be given. If we are fortunate to reach a state of convalescence great care will be needed, as these cases are prone to pass into a state of entero-colitis. Milk should be withheld, and albumin water containing brandy will act nicely until milk, largely diluted, or meat juices, or even scraped raw beef can be borne. General tonics, such as iron, strychnine, hypophosphites, are needed to restore health.

The dyspeptic diarrhea neglected, and cholera infantum with the best of care, will often pass into a chronic form, known as entero-colitis, which is distinguished by pain and tenderness over the colon, some tympanitis all over abdomen, passage of semi-solid stools, mixed with mucus and blood. This state alternates with sudden attacks of acute diarrhea with profuse and watery stools, accompanied by much griping and pain. These cases must be kept absolutely quiet in the cradle, and do better if the cradle is kept out of doors during the daytime. Especial attention must be given to the diet, which should be so regulated that the least possible residue passes over the inflamed intestines. Opium is about the only drug that acts well when given by the mouth. Opium should never be given in cases where the stools are offensive, however much it may seem to be indicated, and on the contrary it will act well in those cases of diarrhea with natural smelling passages. High colonic injections of salt water do good in some cases, cupric arsenite, well diluted, acts favorably at times, as do gallic and tannic acid. If obstinate, nitrate of silver ½ drachm to water one quart acts well, being cautious to follow it by injection of salt water—some give the salt injection first. If there is much tenesmus and some blood, injections into the rectum of starch and laudanum give great relief.

In infants I have never used anything but a soft catheter of linen in giving injections high up, and only introduce it about six inches. By having the syringe bag only slightly elevated and the baby lying on its back the solution will slowly pass up into colon and a large quantity can be received and retained with very little discomfort. Convalescence is slow and the intestines are permanently disabled in some cases.

As I said in the beginning, I have only touched upon a few troubles, and those the ones the most common and familiar, but we oftentimes learn more from criticism and discussion than from the original papers, and thus some good may be accomplished.

